# A tomato MAGIC population reveals candidate genes for leaf dry matter and phenolics, two key traits for stress resilience and climate-smart breeding

**DOI:** 10.3389/fpls.2026.1765593

**Published:** 2026-05-05

**Authors:** Gloria Villanueva, Konstantina Kleftogianni, Ana M. Adalid-Martínez, Andrea Arrones, Oussama Antar, Andrea Solana, Penelope J. Bebeli, Vasileios Papasotiropoulos, Santiago Vilanova, Mariola Plazas, Pietro Gramazio, Jaime Prohens, Leandro Pereira-Dias

**Affiliations:** 1 Instituto de Conservación y Mejora de la Agrodiversidad Valenciana (COMAV), Universitat Politècnica de València, Valencia, Spain; 2 Laboratory of Plant Breeding and Biometry, Department of Crop Science, Agricultural University of Athens, Athens, Greece

**Keywords:** climate-smart varieties, flavonoids, GWAS, phenolic acids, plant fitness, *Solanum lycopersicum* var. *cerasiforme*, *Solanum pimpinellifolium*

## Abstract

Increasing leaf dry matter and phenolic content in tomato has the potential to enhance plant resilience, a key requirement for food security and sustainable agriculture in the context of climate change. However, phenolics accumulation in vegetative tissues remains understudied, as does dry matter accumulation. To address this gap, we leveraged 293 recombinant lines of an eight-way Tomato Multi-Parent Advanced Generation Inter-Cross (ToMAGIC) population to identify candidate genes for leaf dry matter and phenolic content. We found significant variation among lines. Leaf dry matter ranged from 6.90 to 23.20% (CV = 24.84%). Substantial variation was also observed for specialized phenolic metabolites, showing larger differences among lines compared with dry matter. Chlorogenic acid, the most abundant phenolic, ranged from 0.18 to 6.77 g kg^-^¹ FW (CV = 76.1%), followed by rutin (0.026–0.352 g kg^-^¹ FW, CV = 45.1%), quercetin (0.008–0.141 g kg^-^¹ FW, CV = 49.9%), and kaempferol (0.003–0.032 g kg^-^¹ FW, CV = 48.8%). Total phenolic content averaged 3.65 g kg^-^¹ FW, with nearly a 20-fold range (0.47–8.36 g kg^-^¹ FW) (CV = 45.3%). The emergence of highly divergent phenotypes suggests extensive recombination in the population. Traits were highly correlated with each other, with *ρ* ranging from 0.35 to 0.77, supporting the possibility of indirect selection and suggesting common genetic regulation. Likewise, genomic heritability estimates were moderate to low, where chlorogenic acid presented the highest value (*H^2^ =* 0.66) and kaempferol the lowest (*H^2^ =* 0.07). GWAS identified 6 significant associations, explaining 4.50% to 28.24% of the phenotypic variation which, upon further study, enabled the prioritization of candidate genes potentially involved in the control of leaf dry matter and chlorogenic acid content. These findings demonstrate the potential of the ToMAGIC population to facilitate the development of climate-smart tomatoes with improved resilience and reduced input requirements.

## 1 Introduction

Tomato (*Solanum lycopersicum* L.) is a widely cultivated crop, contributing US$116 × 10^9^ to the global economy annually (FAO, 2023). However, its cultivation is undermined by numerous biotic (Panno et al., 2021) and abiotic (Cammarano et al., 2022) stresses. To ensure both food security and the sustainability of agroecosystems, breeding should aim to develop more resilient crops that require fewer inputs.

Dry matter content is a key physiological trait in crops, referring to the proportion of plant biomass made up of structural and functional components such as cellulose, lignin, proteins, carbohydrates, and minerals (Marcelis, 1996). In agricultural science, it is used as a direct indicator of plant fitness, productivity, nutrient density, and water use efficiency (Marcelis, 1996; Cai et al., 2023). By influencing nutrient and water utilization, dry matter content plays a central role in plant growth (Wen et al., 2024), resilience to drought (Blumenthal et al., 2020; Wen et al., 2024), tolerance to herbivory (Blumenthal et al., 2020), and the storage of metabolic reserves (Marcelis, 1996; Poorter et al., 2012). Moreover, it contributes to the development and stability of plant structure (D’Esposito et al., 2019). Despite its importance, the genetic basis of dry matter accumulation in vegetative tissues has received much less attention than dry matter accumulation in reproductive organs (Caruso et al., 2016; D’Esposito et al., 2019).

Phenolic compounds are a diverse group of secondary metabolites highly regarded for the health benefits associated with their consumption. A significant amount of phenolic compounds is present in insoluble forms, being covalently bound to cell wall structural components in the plant food matrix. Therefore, a higher dry matter content, which reflects a greater proportion of structural material, often correlates with increased levels of these bound phenolics (Zhang et al., 2020). These compounds include phenolic acids (e.g., chlorogenic acid, caffeic acid, coumaric acid, and ferulic acid) and flavonoids (e.g., rutin, quercetin, kaempferol). Biosynthesis of phenylpropanoids in fruits has been well characterized and many of the genetic elements involved have been annotated in Solanaceae (Rosa-Martínez et al., 2023). In contrast, phenolic accumulation in vegetative tissues has been less studied, despite their crucial roles in plant organ development (Schijlen et al., 2007; Tohge and Fernie, 2016; Wang et al., 2020), photoprotection (Liu et al., 2018; Righini et al., 2019), and the mediation of plant responses to abiotic stress (Dong and Lin, 2021). Additionally, phenolics contribute substantially to biotic stress tolerance, acting as physical or chemical barriers that limit tissue invasion, as signaling molecules, or as compounds with direct antimicrobial or anti-insect activity (Ramaroson et al., 2022). For instance, increased accumulation of phenolics in leaves was associated with higher resistance to *Pseudomonas syringae* (Dadáková et al., 2020), *Alternaria solani* (Awan et al., 2018), western flower thrips (Leiss et al., 2009), tobacco cutworm (Kundu et al., 2018), silverleaf whitefly (Su et al., 2025), and many other pests and phytopathogens.

Thus, increasing dry matter and phenolic content in tomato leaves has the potential to enhance plant fitness and stress resilience. However, to achieve this, it is essential to understand the mechanisms regulating their biosynthesis and accumulation. So far, the study of genes involved in dry matter and phenylpropanoid accumulation has mostly been conducted using biparental populations, such as introgression lines (ILs), typically derived from crosses between wild and cultivated accessions. For instance, Caruso et al. (2016) and D’Esposito et al. (2019) used a set of ILs, resulting from the cross between *S. pennellii* and *S. lycopersicum*, to dissect residual biomass production mechanisms. Likewise, Minutolo et al. (2013) used ILs 7-3, 10-1, and 12–4 to study the metabolic profiles associated with wild introgressions in the cultivated background. Similarly, Nunes-Nesi et al. (2019) reported QTLs associated with phenylalanine in ILs 3–1 and 9-3-1. These studies provided important insights into the complex nature of these traits and the influence of genotype, environment, and tissue on tomato metabolic profiles. However, they lacked the resolution needed to pinpoint candidate genes controlling phenolic content and were constrained by the limited genetic diversity captured by only two parental lines.

Recently, Multi-parent Advanced Generation Inter-Cross (MAGIC) populations have gained increasing attention, offering higher mapping resolution by inter-mating multiple founders for several generations to generate diverse inbred lines with mosaic genomes (Cavanagh et al., 2008; Arrones et al., 2020; Scott et al., 2020). We developed a MAGIC population with 354 recombinant lines (ToMAGIC) by inter-crossing four weedy *S. l.* var. *cerasiforme* (SLC) with four wild *S. pimpinellifolium* (SP) accessions (Arrones et al., 2024). These materials encompass the Andean variability lost during the domestication process in Mesoamerica and are considered reservoirs of untapped genetic variability for traits related to plant and inflorescence architecture, leaves, flowers, fruits, pest and disease resistance, and tolerance to abiotic stresses (Blanca et al., 2015; Gramazio et al., 2020; Martínez-Cuenca et al., 2020). The ToMAGIC population has a high degree of homozygosity, an absence of genetic structure, and balanced representation of the founder genomes (Arrones et al., 2024). In a recent publication, we used the ToMAGIC population as a proof-of-concept of high-precision mapping by validating previously identified genes and identifying new candidates associated with several plant and fruit traits (Arrones et al., 2024). This population could be a valuable resource for dissecting the genetic basis of complex traits, identifying novel alleles, and ultimately introgressing beneficial alleles to develop improved tomato varieties.

In the framework of our current research, we leveraged the ToMAGIC population to identify putative genes controlling dry matter and phenolic accumulation of young leaves. The large variability within the population resulted in different accumulation levels. Recombinant lines, especially those with high dry matter content and/or high phenolic levels, are of particular interest to breeders. Their sexual compatibility with cultivated tomato makes them potential donors for introgression of favorable alleles (Blanca et al., 2015). An additional outcome of our study is that the use of a core subset of the ToMAGIC population can contribute to tomato genetics research without the need to use the entire population. Overall, this work is highly relevant for advancing the development of climate-smart tomato varieties with reduced input requirements, thereby contributing to sustainable agriculture and resource-efficient crop production.

## 2 Material and methods

### 2.1 ToMAGIC population construction

The interspecific tomato MAGIC population (ToMAGIC) was developed by manually inter-crossing four weedy *Solanum lycopersicum* var. *cerasiforme*, i.e., BGV007931 (SLC1), LA2251 (SLC2), PI487625 (SLC3), and BGV006769 (SLC4), and four wild *Solanum pimpinellifolium* founder accessions, i.e., BGV007145 (SP1), BGV006454 (SP2), BGV015382 (SP3), and BGV013720 (SP4) (Gramazio et al., 2020; Martínez-Cuenca et al., 2020; Arrones et al., 2024). Founder lines were intercrossed following a “funnel” approach, generating first four F_1_ hybrids, then four double hybrids, and finally 112 IC_1_ individuals. Subsequent intercrossing phases followed a chain-pollination scheme, resulting in 232 IC_2_ and 481 IC_3_ individuals. IC_3_ individuals were selfed for five generations by single-seed descent, resulting in a final population of 354 S5 recombinant inbred lines with a low level of heterozygosity (~3%) (Arrones et al., 2024). For the present work only 293 lines were available for testing (i.e., enough seed stock).

### 2.2 Plant material and growth conditions

Two hundred and ninety-three ToMAGIC lines were germinated in seedling trays containing Humin Substrat No. 3 (Neuhaus, Klasmann-Deilmann, Germany) in a climate chamber under a 16 h light/8 h dark photoperiod, with temperatures of 25 °C during the light period and 18 °C during the dark period. At the five-true-leaf stage, two plantlets per genotype were transplanted into 1.5 L thermoformed pots filled with Humin Substrat No. 3 (Neuhaus, Klasmann-Deilmann, Germany), composed of sphagnum frozen black peat and sphagnum white peat enriched with 1 kg m^-3^ of 14-10-18 (N-P-K) fertilizer (85% organic matter, pH 6, electrical conductivity of 35 mS m^-1^, and water retention of 75%). The two plants per genotype were grown concurrently in the same pollinator-free, climate-controlled glasshouse at the Universitat Politècnica de València (Valencia, Spain), at 15-27 °C, and were watered manually every 1–3 days according to plant needs. The experiment followed a randomized complete block design with two blocks, each containing one plant per genotype; thus, the two plants per genotype represented two biological replicates (*n* = 2).

### 2.3 Dry matter and phenolics quantification

For each of the 293 ToMAGIC lines, leaves were sampled from two independent plants representing biological replicates (*n* = 2) at anthesis of the first flower of the second truss. For each plant, 5 g of leaf tissue was obtained by pooling multiple leaves randomly collected from across the plant during the morning hours, excluding stems and leaf petioles. Tissue was composited within plants, but not across plants. Samples were lyophilized using a VirTis Genesis lyophilizer (SP Scientific, Warminster, PA, USA) and ground into a fine powder using a coffee grinder. Dry matter (%) was calculated as (freeze-dried weight/fresh weight) × 100.

To study phenolic accumulation in young leaves, a representative subset of 130 lines, each represented by two biological replicates (*n* = 2), was selected among the 293 lines using *ShinyCore* software (Kim et al., 2023). *ShinyCore* ensures the preset minimum coverage (proportion of marker alleles in the entire collection that are retained in the core subset), and it then selects lines carrying the rarest allele at each marker. Thus, the resulting subset better captures genetic diversity and distance (Kim et al., 2023). The program was run to capture at least 90% coverage.

Phenolic acids (i.e., chlorogenic acid, caffeic acid, coumaric acid, and ferulic acid) and flavonoids (i.e., rutin, quercetin, and kaempferol) were extracted using 0.1 g of freeze-dried leaf homogenate, as described in Rosa-Martínez et al. (2021). One aliquot of this extract was reserved for hydrolysis by adding 3 M HCl (2:1, v/v) for 1 h at 95 °C, to free the flavonoids aglycones from their attached sugar moieties. Samples were analyzed using a Shimadzu SCL-40 system (Shimadzu Corporation, Japan) coupled with a diode array detector and equipped with a Brisa LC2 C18 column (3 μm, 150 × 4.6 mm; Teknokroma, Spain). Two gradient elution programs were used to analyze phenolic acids (EP1) and aglycones (EP2), as described in Guijarro-Real et al. (2019). The same mobile phase, consisting of solvent A (0.1% formic acid in ultrapure water) and solvent B (0.1% formic acid in methanol), was used for both elution programs. The flow rate was 1 mL min^-1^ (EP1) and 0.8 mL min^-1^ (EP2). The injection volume was 10 µL and the column temperature was maintained at 30 °C in both methods. Peaks were detected at a fixed wavelength of 320 nm (EP1) and 360 nm (EP2). Peak identification was based on comparison of retention times and UV spectra with those of standard compounds. Quantification was performed using external calibration curves generated from serial dilutions of HPLC-grade standards. Only peaks above the detection limit (LOD), defined as a signal-to-noise ratio of 3:1, were considered for quantification.

Total phenolics were extracted using 0.1 g of freeze-dried leaf homogenate from a subset of 130 core lines, each represented by two biological replicates (*n* = 2), and spectrophotometrically determined at 750 nm according to the Folin-Ciocalteu procedure using a Model 550 Microplate Reader (Bio-Rad, CA, USA), as described in Rosa-Martínez et al. (2021). For each biological replicate, the extract was measured in three independent wells (*n* = 3), which were considered technical replicates. Statistical analyses were performed using plant-level values calculated as the mean of the technical replicates. Gallic acid was used as the reference standard (Sigma-Aldrich, MO, USA).

### 2.4 Statistical analysis

Analysis of variance (ANOVA) was performed using the *Stats* package (R Core Team, 2024). Spearman’s rank correlations (*ρ*) were calculated with the *PerformanceAnalytics* package (Brian G. Peterson et al., 2024) using the mean values of the lines and considering only the 130 lines subset. Plots were generated with *ggplot2* (Wickham, 2016). The genomic relationship matrix (GRM) was constructed using the VanRaden method (VanRaden, 2008) from the quality-controlled SNP dataset described in the GWAS section below. To ensure positive semidefiniteness, a small constant was added to the diagonal of the GRM. The GRM was then matched to the phenotypic data, and its inverse was computed for subsequent analyses. Variance components were estimated by fitting a mixed model using the *sommer* R package (Covarrubias-Pazaran, 2016), where genotypes were fitted as a random effect with the genomic covariance structure, and blocks were fitted as an additional random effect. Residuals were assumed to be independently and identically distributed. Genomic heritability was estimated using the Cullis method (Cullis et al., 2006), which accounts for the prediction error variance of the BLUPs. Specifically, Cullis heritability was computed as $H_{Cullis}^{2}=1-\frac{Mean\;prediction\;error\;variance\;of\;the\;BLUPs}{2\;\cdot \;\sigma_{g}^{2}}$, where $\sigma_{\text{g}}^{2}$ is the estimated genetic variance scaled by the average diagonal of the GRM. The prediction error variance was obtained from the mixed model output.

### 2.5 Genome-Wide Association Study (GWAS)

A panel of 6,488 highly informative SNPs generated with Single Primer Enrichment Technology (SPET), covering around 16.91% of the total annotated genes and averaging 8.51 markers per Mb (Barchi et al., 2019; Arrones et al., 2024), was used for GWAS. Raw SNPs generated by SPET were first filtered based on sequencing depth (coverage ≥ 10), genotype quality score (GQ ≥ 20). After that, monomorphic sites were removed using BCFtools (Danecek et al., 2021). Subsequently, further filtering was performed using TASSEL v5.0 (Bradbury et al., 2007) to retain high-confidence variants, applying the following criteria: minor allele frequency (MAF) > 0.01, missing genotype rate < 10%, and maximum marker heterozygosity < 0.7. Missing genotypes were imputed using the linkage disequilibrium k-nearest neighbor imputation method (LD-KNNi) implemented in TASSEL (Money et al., 2015). A high SNP density was previously reported in gene-rich regions outside the pericentromeric regions for this marker set in this population (Arrones et al., 2024).

GWAS was performed using GAPIT v3.5 (Wang and Zhang, 2021) with the Multiple analysis option enabled and the following models: Compressed Mixed Linear Model (CMLM), General Linear Model (GLM), Mixed Linear Model (MLM), Multiple Loci Mixed Model (MLMM), and Bayesian-information and Linkage-disequilibrium Iteratively Nested Keyway (BLINK) (Price et al., 2006; Yu et al., 2006; Zhang et al., 2010; Segura et al., 2012; Huang et al., 2018). Trait distributions were evaluated for normality using the Shapiro–Wilk test. Although some traits showed deviations from normality, no transformations were applied because ANOVA and mixed linear models used for GWAS are generally robust to moderate departures from normality, particularly with balanced designs and adequate sample sizes. Model residuals were inspected to verify that no severe violations of normality or homoscedasticity were present, supporting the use of raw phenotypic values for subsequent analyses. QQ plots were used to compare model fit and assess potential inflation of test statistics. The use of multiple GWAS models increases the robustness of marker–trait associations by accounting for different statistical assumptions and strategies for controlling population structure and kinship. SNPs consistently identified across several models are less likely to be false positives and therefore can be considered more reliable candidate loci. Accordingly, priority was given to SNPs identified by the model considered most robust in this context (i.e., BLINK) (Huang et al., 2018; Wang and Zhang, 2021). Although FarmCPU provides good statistical power, it may be sensitive to pseudo-QTN selection, marker density, and sample size (Huang et al., 2018). For this reason, it was not included. The proportion of phenotypic variance explained (PVE) by significant markers was calculated using the BLINK model.

Best linear unbiased estimates (BLUEs) of line effects for the traits were obtained using the *sommer* R package (Covarrubias-Pazaran, 2016). A linear mixed model was fitted with genotype as a fixed effect and block as a random effect, while residuals were modeled as independently and identically distributed. The intercept was set to zero to obtain BLUEs for each line. Population structure (Principal Components, PCs) and the kinship matrix (K) were inferred directly from the genotype data using the GAPIT pipeline (Wang and Zhang, 2021). The General Linear Model (GLM) included population structure but did not account for kinship, whereas BLINK does not explicitly incorporate a kinship matrix, instead controlling confounding through linkage disequilibrium (LD) among markers. The first three PCs were included as covariates to account for population structure. The kinship matrix was calculated using the VanRaden (2008) method implemented in GAPIT. Correction for multiple testing was performed using the Bonferroni method with a significance threshold of *α* = 0.05.

SNPs exceeding the specified threshold were considered significantly associated with the traits under study. The most significant SNPs were used to delimit a fixed physical window of ±100,000 bp around them to mine for candidate genes previously reported to be involved in the trait, or belonging to gene families implicated in it. The genes underlying the fixed interval were retrieved from the Heinz 1706 SL4.0 tomato reference genome annotation (Hosmani et al., 2019). Finally, SnpEff v4.2 (Cingolani et al., 2012) was used to predict the impact of the variants in the candidate genes.

The Tomato Functional Genomics Database was used to assess gene tissue specificity and gene expression (Fei et al., 2011) when no relevant information was found in the literature. Candidate genes were queried against the processed RNA-seq data deposited in the database. Normalized expression values (RPKM) were examined, with preference given to *S. pimpinellifolium* or *S. lycopersicum* var. *cerasiforme* datasets. We considered genes with RPKM ≥ 1 as expressed and RPKM ≥ 3 as moderately to strongly expressed. Tissue relevance was assessed by comparing expression in young leaves with the mean expression across the remaining tissues. Genes with a young leaf/other tissues mean expression ratio ≥ 1 were considered highly relevant to young leaves, whereas genes with a ratio between 0.3 and <1 were considered relevant.

## 3 Results

### 3.1 Analysis of variance and variation observed

The ANOVA revealed a significant effect of genotype on all traits except for rutin and kaempferol (**Table 1**). The results indicated large differences among lines (**Supplementary Data Sheet 1**), with phenolic traits generally showing more variation than dry matter.

**Table 1:** Mean, standard deviation (SD), minimum (Min), maximum (Max), variation coefficient (CV), *p*-value, and genomic heritability (*H^2^*_Cullis_) for dry matter and polyphenols measured in the tomato ToMAGIC population’s young leaves.

Traits^1^	Mean	SD	Min	Max	CV (%)	*P*-value*	*H^2^*_Cullis_
Dry matter (%)	13.61	3.38	6.90	23.20	24.84	0.000	0.62
Chlorogenic acid (g kg^-^¹ FW)	2.12	1.61	0.18	6.77	76.05	0.000	0.66
Rutin (g kg^-^¹ FW)	0.147	0.066	0.026	0.352	45.14	0.063 ^ns^	0.39
Quercetin (g kg^-^¹ FW)	0.059	0.030	0.008	0.141	49.90	0.006	0.43
Kaempferol (g kg^-^¹ FW)	0.012	0.006	0.003	0.032	48.80	0.709 ^ns^	0.07
Total phenolic content (g kg^-^¹ FW)	3.65	1.65	0.47	8.36	45.25	0.020	0.56

^1^ Dry matter was obtained for 293 lines whereas the rest of traits were measured for a subset of 130 lines selected with *ShinyCore* package. *ns indicated no significant differences among lines.

On average, ToMAGIC lines had a mean leaf dry matter content of 13.61% (**Table 1**), with line 426 (23.20%) showing the highest mean value, followed by lines 448 and 586 (22.90 and 22.40%, respectively) (**Supplementary Data Sheet 1**). Chlorogenic acid content averaged 2.12 g kg^-^¹ FW. Lines 763 and 782 showed the highest mean values in the entire subset (6.77 and 5.52 g kg^-^¹ FW, respectively) (**Supplementary Data Sheet 1**). Rutin averaged 0.147 g kg^-^¹ FW with line 763 showing the highest mean value (0.352 g kg^-^¹ FW), followed by line 839 with 0.329 g kg^-^¹ FW (**Supplementary Data Sheet 1**). Quercetin averaged 0.059 g kg^-^¹ FW (**Table 1**), with lines 776 (0.141 g kg^-^¹ FW), 756 (0.136 g kg^-^¹ FW), and 761 (0.133 g kg^-^¹ FW) showing the highest quercetin content (**Supplementary Data Sheet 1**). Kaempferol was the least abundant metabolite in this experiment, averaging 0.012 g kg^-^¹ FW (**Table 1**). The highest value was observed for lines 776 and 430 (0.032 g kg^-^¹ FW), with lines 859, 832, and 724 following closely (0.025 g kg^-^¹ FW) (**Supplementary Data Sheet 1**). Total phenolic content, according to the Folin-Ciocalteu method, averaged 3.65 g kg^-^¹ FW across the evaluated lines (**Table 1**). Line 796 (8.36 g kg^-^¹ FW) had the highest mean content, with lines 413 (8.17 g kg^-^¹ FW) and 782 (7.58 g kg^-^¹ FW) following closely (**Supplementary Data Sheet 1**).

Coumaric and ferulic acids were not detected in young tomato leaves. Moreover, caffeic acid was below the detection limit in most lines, although a few exhibited low levels of accumulation in the leaves (mean < 0.010 g kg^-^¹ FW). For this reason, coumaric, ferulic, and caffeic acids were excluded from further analysis.

### 3.2 Correlations and heritability

**Figure 1:**
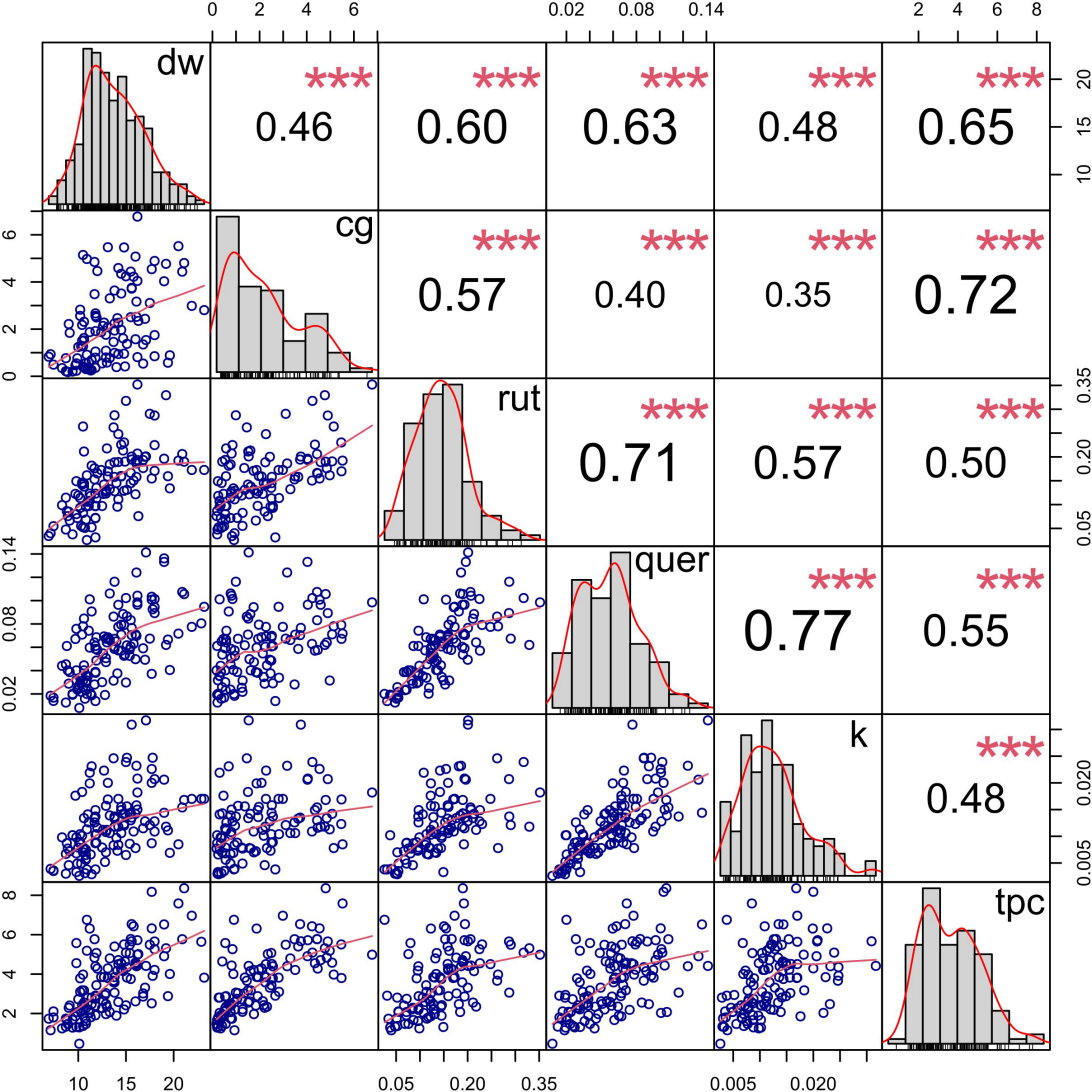
Spearman’s rank correlations (upper diagonal; *ρ*) among the traits dry matter (dw), chlorogenic acid (cg), rutin (rut), quercetin (quer), kaempferol (k), and total phenolic content (tpc). Statistically significance is indicated with asterisks (*** *p< 0.001*). Phenotype frequency distribution histogram for the ToMAGIC lines (diagonal). Bottom diagonal shows scatter plots for each pair of variables, helping visualize their relationships.

Spearman’s rank correlations revealed that all traits were positively and significantly correlated with each other (**Figure 1**). Dry matter (dw) showed high correlations (*ρ* = 0.46-0.65) with all quantified metabolites (**Figure 1**). Its strongest correlation (*ρ* = 0.65) was with total phenolic content (tpc). Likewise, chlorogenic acid (cg) and the flavonoids were strongly correlated with tpc, with correlation coefficients ranging from *ρ* = 0.48 for kaempferol to *ρ* = 0.72 for chlorogenic acid. The highest correlation among phenolic compounds (*ρ* = 0.77) was observed between quercetin and kaempferol. Rutin, which is the main precursor of these two flavonols, was highly correlated with both (*ρ* = 0.71 and 0.57, respectively). Conversely, the lowest correlation (*ρ* = 0.35) was observed between chlorogenic acid and kaempferol.

Genomic heritability estimates (*H^2^*) (Cullis et al., 2006) were moderate to low (**Table 1**). Chlorogenic acid presented the highest estimate (*H^2^* = 0.66) whereas kaempferol displayed the lowest (*H^2^* = 0.07). Dry matter and total phenolic content had the second- and third-highest *H^2^* values (*H^2^* = 0.62 and 0.56, respectively), followed by quercetin (*H^2^* = 0.43) and finally rutin (*H^2^* = 0.39) (**Table 1**). For all traits, phenotypic variation exceeded genotypic variation.

### 3.3 Genome-Wide Association Study (GWAS)

Overall, GWAS models showed a good fit to the data, as indicated by the quantile-quantile plots (**Supplementary Data Sheet 2**). For dry matter and total phenolic content, GLM displayed more *p*-values deviating from the null hypothesis of no association, compared with the other models tested, suggesting higher probability of false-positives when using this model (**Supplementary Data Sheet 2**). BLINK (the most statistically robust method in this context) was able to uncover more significant associations (**Table 2**; **Supplementary Data Sheet 3**).

**Table 2:** Significant associations found for dry matter and chlorogenic acid in the tomato ToMAGIC population.

Trait	Model	Chr	SNP (bp)	Variance explained^1^	Closest candidate gene*	Alternative candidate gene/s*
Dry matter	GLM, BLINK	Chr01	81,163,356	6.71%	Gibberellin Receptor (*Solyc01g098390*)	LOB Protein 20 (Solyc01g098220*), FRIGIDA-like Protein (Solyc01g098240*), Sugar Facilitator 1 (Solyc01g098490*), Sugar Facilitator 5 (Solyc01g098500**)
Dry matter	BLINK	Chr04	5,092,132	5.76%	GRAS Transcription Factor (Solyc04g014830**)	MYB/SANT-like Protein (Solyc04g014855*)
Dry matter	BLINK	Chr05	61,754,534	4.50%	Ethylene Response Factor 4 (*Solyc05g052030*)	Major Facilitator Superfamily Protein (Solyc05g051920**)
Dry matter	GLM, MLMM, BLINK	Chr05	63,985,064	17.13%	Sulfate Transporter (Solyc05g054740**)	Zinc Finger Protein (Solyc05g054650*), Beta-hexosaminidase (Solyc05g054710***), HERK 1 (Solyc05g054860*)
Dry matter	MLMM, BLINK	Chr12	2,380,493	7.54%	WD40 (*Solyc12g009030*)	Cytokinin Dehydrogenase 5 (Solyc12g008920**), MAP Kinase 1 (*Solyc12g009020*)
Chlorogenic acid	All	Chr10	1,106,268	28.24%	Protein Kinase (*Solyc10g006790*)	Auxin-responsive GH3 Protein (Solyc10g006610**, Solyc10g006615**, Solyc10g006630*), bHLH Transcription Factor 153 (Solyc10g006640*), Quinone Reductase (Solyc10g006650*), THESEUS 1 (Solyc10g006870*)

^1^ Calculated using the BLINK model; * Indicates the number of high-impact mutations in the candidate’s genes coding sequences detected in the parents of the ToMAGIC population using SnpEff (**Supplementary Data 4**).

For each association information is provided regarding the GWAS model through which it was identified, the chromosome and physical position of the significant SNP, the variance explained by it, the closest candidate gene, and alternative candidate genes that are located within the ±100 kb physical window around the significant SNP.

**Figure 2:**
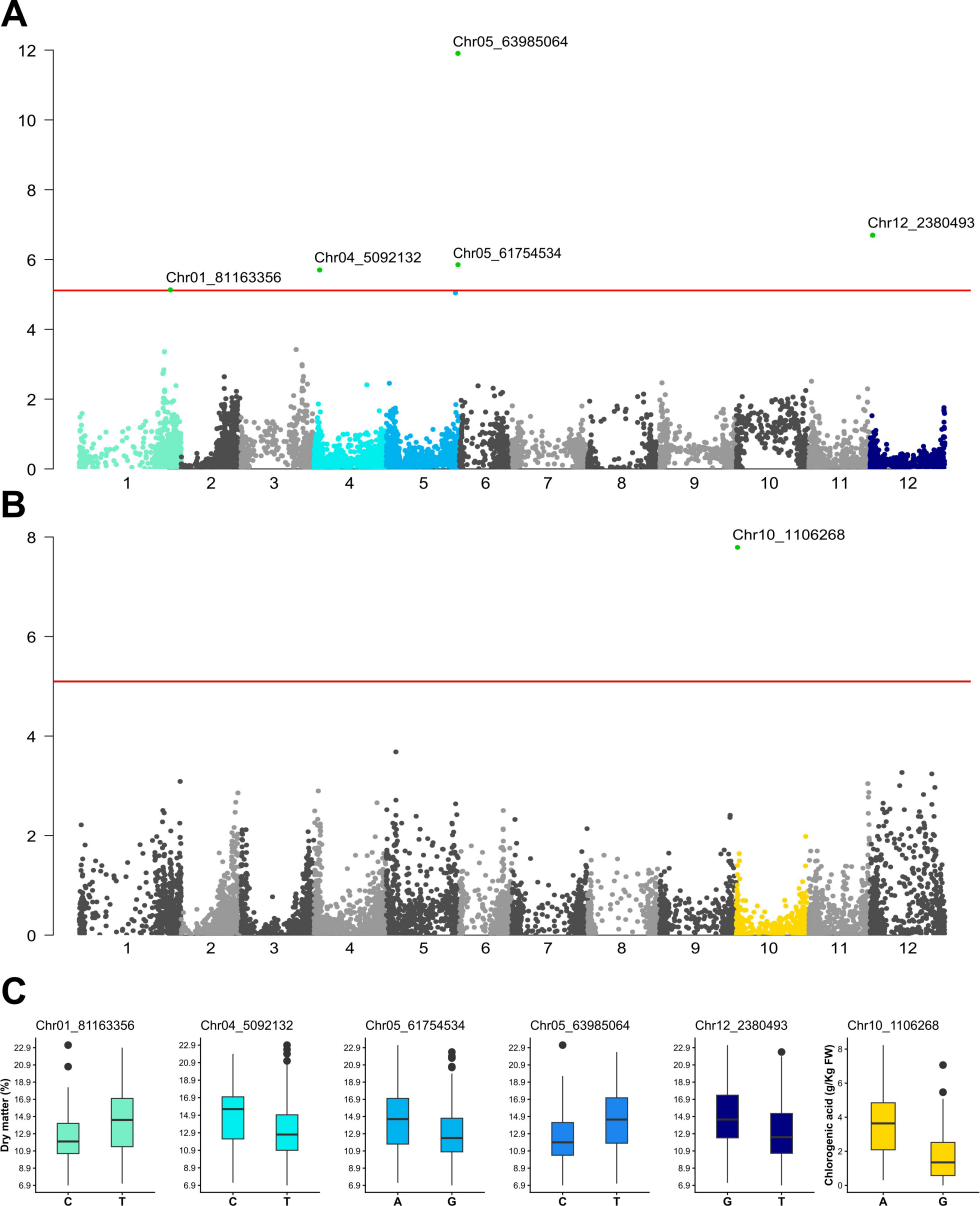
**(A)** Manhattan plot showing all significant SNPs associated with dry matter content. **(B)** Manhattan plot showing significant SNP associated with chlorogenic acid content. The solid red line represents the Bonferroni threshold (*-log_10_ (p)* > 5.11); dots above this line indicate statistically significant associations between genotype and phenotype. BLINK trait-specific Manhattan plots for other traits can be found in **Supplementary Data Sheet 3**. **(C)** Boxplots showing the allelic effects (*p* < 0.05) of the six significant SNPs associated with dry matter and chlorogenic acid content. Colors in **(A–C)** correspond to the same chromosomes to facilitate comparison between SNP locations and allelic effects.

Dry matter content was associated with five SNPs above the Bonferroni threshold on chromosomes 1, 4, 5, and 12 (**Figure 2A**; **Table 2**). The first locus was detected at 81,163,356 bp on chromosome 1 by the GLM and BLINK models. Lines carrying the T allele showed significantly higher dry matter content than those carrying the reference C allele (14.41% vs 12.40%) (**Figure 2C**). This SNP explained 6.71% of the phenotypic variance.

A second association was identified on chromosome 4 at 5,092,132 bp by BLINK. Lines carrying the C allele showed higher dry matter content than those carrying the reference T allele (14.84% vs 13.21%). This locus explained 5.76% of the phenotypic variance for this trait (**Figure 2A, C**; **Table 2**).

On chromosome 5, two significant associations were identified (**Figure 2A**; **Table 2**). The first locus, located at 61,754,534 bp, detected by BLINK, explained 4.50% of the phenotypic variance. Lines carrying the reference A allele showed higher dry matter content than those carrying the G allele (14.55% vs 12.96%) (**Figure 2C**). The second locus, located at 63,985,064 bp and detected by GLM, MLMM, and BLINK, explained 17.13% of the phenotypic variance for this trait. Lines carrying the reference T allele showed significantly higher dry matter content than those carrying the C allele (14.59% vs 12.35%), corresponding to a 15.39% increase.

The final dry matter association was identified on chromosome 12 at position 2,380,493 bp by MLMM and BLINK. This SNP explained 7.54% of the phenotypic variance, with lines carrying the G allele showing higher dry matter content than those carrying the reference T allele (14.91% vs 13.06%) (**Figure 2A, C**; **Table 2**).

For chlorogenic acid content, one significant SNP was detected consistently across all models on chromosome 10 at 1,106,268 bp (**Figure 2B**). This SNP explained 28.24% of the phenotypic variance. Lines carrying the reference A allele showed 50.42% higher chlorogenic acid content than that of lines carrying the G allele (3.61 vs 1.79 g kg^-^¹ FW) (**Figure 2C**; **Table 2**).

Candidate genes were prioritized based on their physical proximity to significant SNPs within a ±100 kb window and relation with the trait. Prioritization also considered the presence of SnpEff-predicted high-impact variants, including frameshift mutations, stop-gained variants, splice-site alterations, and premature start codon gains, segregating among the eight ToMAGIC parental lines (**Supplementary Data Sheet 4**). Functional annotations related to plant development, transcriptional regulation, metabolite transport, and hormone signaling were also considered to support the potential biological relevance of these genes.

The candidate gene closest to the chromosome 1 locus was a *Gibberellin receptor* (*Solyc01g098390*). Other candidate genes included *LOB domain-containing protein 20* (*Solyc01g098220*), a *FRIGIDA-like protein* (*Solyc01g098240*), and two *Sugar Facilitator Proteins* (*Solyc01g098490* and *Solyc01g098500*) (**Table 2**). These genes harbor high-impact mutations, including stop-gained, premature start codon gain, and frameshift variants (**Supplementary Data Sheet 4**).

The association found on chromosome 4 was located within the exonic sequence of a GRAS transcription factor (**Table 2**). The missense variant may have a non-disruptive but potentially functional effect on the encoded protein. Moreover, this candidate harbored two other high-impact mutations (frameshift variants) in its coding sequence in two *S. lycopersicum* var. *cerasiforme* parental lines (**Supplementary Data Sheet 4**), which could contribute to the observed variation among lines. However, we found other plausible candidates in this physical window, namely a *MYB/SANT-like Protein* (*Solyc04g014855*) (**Table 2**). This gene carried a frameshift mutation (**Supplementary Data Sheet 4**).

On chromosome 5, the candidate gene closest to SNP 61,754,534 was *Ethylene Response Factor 4 (ERF4)* (*Solyc05g052030*) which lacked high-impact mutations (**Table 2**). An alternative candidate gene was the *Major Facilitator Superfamily protein* (*Solyc05g051920*), which carried two frameshift mutations, suggesting a possible role in dry matter variation (**Supplementary Data Sheet 4**). The association at 63,985,064 bp was in the intronic region of *Sulfate Transporter* (*Solyc05g054740*) (**Table 2**). This gene carried a splice acceptor variant in two parental lines (**Supplementary Data Sheet 4**), potentially leading to loss of function or aberrant proteins. Near this position, a *Zinc Finger Protein* (*Solyc05g054650*), a *β-hexosaminidase* (*Solyc05g054710*), and a *HERK 1 Protein Kinase* (*Solyc05g054860*) each showed at least one high-impact variant that may be segregating in the population and contributing to trait variation (**Supplementary Data Sheet 4**). Due to their physical proximity, these associations may represent the same underlying locus. Under this assumption, *OBV* (*Solyc05g054030*) emerges as a strong candidate gene, in addition to the ones mentioned above.

The chromosome 12 SNP was located within the coding sequence of *WD40 Transcription Factor* (*Solyc12g009030*) and corresponded to a missense variant (**Table 2**). No high-impact mutations were predicted for this gene. The upstream-located *Cytokinin dehydrogenase 5* (*Solyc12g008920*) was also considered a strong candidate because it carried splice-site variants in several ToMAGIC parents (**Supplementary Data Sheet 4**), in addition to its known role in plant development.

Finally, the locus on chromosome 10 was associated with chlorogenic acid content. The closest candidate gene was *Protein kinase* (*Solyc10g006790*) which presented no high-impact variants (**Table 2**). In addition, *Auxin-responsive GH3 Family Proteins* (*Solyc10g006610*, *Solyc10g006615*, and *Solyc10g006630*), *bHLH Transcription Factor 153* (*Solyc10g006640*), *Quinone reductase* (*Solyc10g006650*), and *THESEUS 1 Protein Kinase* (*Solyc10g006870*) were identified as additional candidates. These encompass at least one high-impact mutation among the ToMAGIC parental lines, potentially affecting the accumulation of this phenolic in the tomato leaves (**Supplementary Data Sheet 4**).

## 4 Discussion

We present the first quantification of dry matter and polyphenols content, two important traits for stress resilience and adaptation to climate change, in leaves from the tomato MAGIC population (ToMAGIC). This type of population has been used to map QTLs, dissect complex traits, identify causal polymorphisms, and support participatory breeding schemes to combine agronomic performance with local adaptation under low-input or organic conditions (Pascual et al., 2015; Campanelli et al., 2019; Arrones et al., 2024). MAGIC populations are developed through multiple rounds of intercrossing and selfing, thereby generating a high degree of recombination, reducing linkage block size and improving mapping accuracy (Cavanagh et al., 2008). In addition, they minimize genetic structure and balanced representation of the founder genomes, thus reducing spurious associations (Arrones et al., 2020). Moreover, the combination of multiple founders provides higher genetic and phenotypic diversity, thus increasing the number of QTLs segregating in the population (Scott et al., 2020). These advantages coupled with powerful models have dramatically increased the statistical power of GWAS (Tibbs Cortes et al., 2021).

Our results revealed the broad variation present in the ToMAGIC population for dry matter and phenolic content. Other authors have observed that QTL directionalities reflect differences among parental lines, often leading to a considerable degree of transgressive behavior and resulting in extreme phenotypes (Pascual et al., 2015; Nunes-Nesi et al., 2019; Arrones et al., 2024). The positive correlations among the evaluated traits suggest that improving one trait could potentially improve others, as similarly reported for tomato fruit composition, where comparable correlations among traits have been observed (Bhandari et al., 2016; Alhaithloul et al., 2021; Jin et al., 2022). The genomic heritability estimates revealed moderate to low values. These findings suggest that while some traits may be more amenable to selection, others, such as kaempferol, may require more complex strategies, as phenotypic variation is largely influenced by environmental factors (Alseekh et al., 2015; Rosa-Martínez et al., 2023). Thus, a tiered selection strategy using dry matter as a primary screening trait could reduce phenotyping effort, while complementary phenotyping or marker-assisted selection could support more reliable improvement of phenolic composition.

Works by other researchers on *S. pennellii* ILs uncovered substantial variation in dry biomass accumulation. Caruso et al. (2016) showed that introgressions on chromosomes 2 and 4 produced high levels of dry residual biomass (leaves, shoots, and stems), whereas introgression on chromosome 3 was associated with lower dry residual biomass accumulation. These authors were able to identify ILs 2–5, 2–6, 6–3, 7–2, 10–2, and 12–4 combining good fruit production and residual biomass accumulation (Caruso et al., 2016). Likewise, de Oliveira Silva et al. (2018) identified five QTLs associated with higher shoot dry matter accumulation on chromosomes 2, 7, 9, 10, and 12, and eight associated with lower accumulation on chromosomes 1, 3, 4, 7, and 9. Our results also suggest that the distal region of chromosome 1 plays an important role in regulating this trait, consistent with findings for *S. pennellii* ILs 1–4 and 1-4-18. Both studies found associations on chromosomes 4 and 12, as we did, but our associations mapped to telomeric regions, whereas theirs mapped to more proximal or interstitial regions. The differences in plant material analyzed (leaves vs whole aerial biomass) and genetic background (*S. pennellii* vs *S. pimpinellifolium/cerasiforme*) among studies may explain these results. Furthermore, our results confirm that dry matter accumulation is a complex trait controlled by numerous QTLs (de Oliveira Silva et al., 2018; D’Esposito et al., 2019).

Dry matter content is influenced by genes regulating cell division, cell wall synthesis, metabolite accumulation, and carbon metabolism (D’Esposito et al., 2019). Transcription factors orchestrate a wide range of biological processes that directly and indirectly influence dry matter. *SlGRAS14* (*Solyc04g014830*) was associated with dry matter content. The GRAS family plays crucial roles in plant growth and development. The high sequence similarity with *AtSCL13* (*SCARECROW-like 13*), which is associated with phytochrome A and B signaling, suggests that *SlGRAS14* may have a similar function, possibly by controlling hypocotyl elongation (Huang et al., 2015). Likewise, C_2_H_2_-type Zinc Finger Proteins (ZFPs), such as *OBV* (*Solyc05g054030*), influence leaf development and plasticity in response to growth irradiance (Lu et al., 2021). Strikingly, this gene is located between the two QTLs found for dry matter content in chromosome 5. Because these associations are physically close to each other, we could not rule out the possibility that they may point to the same candidate region, despite the presence of strong candidate genes near the peak SNPs. The ZFP (*Solyc05g054650*) proposed as an alternative candidate has also been reported to be expressed in tomato leaves with a modest response to biotic and abiotic stresses (Zhao et al., 2020), but seems to have a more prominent role in root tissues (Hu et al., 2019).

Membrane transporters directly affect carbon partitioning, source–sink relationships, and ultimately dry matter accumulation. *Sugar facilitator Protein 1* and *5* (SFPs) prioritized in this study could mediate the transport of carbohydrates into young leaves to feed cell division and wall formation, thereby contributing to increased dry matter content. Supporting this idea, *SlSFP5* is expressed in leaves, as well as at the later stages of fruit development (Reuscher et al., 2014). A similar role would be expected for the *Major Facilitator Protein* gene associated with dry matter on chromosome 5 in our work, although proteins in this family have broader substrate specificity as reported in studies in Arabidopsis and tomato (Reuscher et al., 2014; Calafiore et al., 2019; Niño-González et al., 2019).

Our findings indicate that chlorogenic acid is the most abundant phenolic compound in ToMAGIC leaves. These results align with those reported by Larbat et al. (2014), although the mean value for the ToMAGIC population was approximately half that reported for *S. lycopersicum* cherry varieties, such as Micro-Tom, Red Robin, Tiny Tim, and Florida Basket. In contrast, our values were higher than those observed in *S. pennellii* ILs 7-3, 10-1, and 12-4, as reported by Minutolo et al. (2013). These authors found no significant variation in chlorogenic acid content, despite IL 12–4 containing a 4-coumarate:CoA ligase gene in the introgressed fragment. They instead identified rutin as the most abundant compound in the leaves, with concentrations comparable to ours. However, Larbat et al. (2014) reported rutin concentrations 20- to 60-fold higher than those observed in our study. Similarly, the concentrations of kaempferol in their study were about 20 times higher than ours. In contrast, we found quercetin to be more abundant than kaempferol (approximately sixfold). Finally, while Minutolo et al. (2013) detected both ferulic and caffeic acids, we found ferulic to be below the limit of detection and caffeic acid was detected at low concentrations for only one third of the lines. The low values and the inconsistency between replicates led us not to investigate their genetic basis, although it may be worth selecting caffeic acid-enriched plants since caffeic acid and related compounds are defense-associated molecules (Dadáková et al., 2020; Mughal et al., 2024). Hence, differences in genetic backgrounds, developmental stages, and experimental conditions can significantly influence metabolic profiles, which likely contributed to the observed differences. Nonetheless, these findings underscore the considerable variation within the tomato gene pool, especially in ToMAGIC, which offers potential for developing climate-smart lines that are richer in phenolic acids or flavonoids and more resilient to stressors (Leiss et al., 2009; Kundu et al., 2018; Righini et al., 2019; Su et al., 2025).

Phenylpropanoids are synthesized through a well-defined pathway, but their regulation involves a diverse set of regulatory elements, post-translational modifiers, and cellular transporters (Pucker and Selmar, 2022; Rosa-Martínez et al., 2023), which is reflected in the panel of candidate genes prioritized in this study. In a study of tomato leaf metabolism, Nunes-Nesi et al. (2019) reported two QTLs associated with phenylalanine at the proximal region of chromosome 3 (IL3-1) and at the distal region of chromosome 9 (IL9-3-1) in four-week-old non-flowering *S. pennellii* ILs. These QTL associations were not replicated in our work, which may reflect differences in environmental conditions, developmental stage, marker density, and the complex genetic architecture of these traits, rather than limited diversity in the ToMAGIC founders. Chromosome 10 was instead associated with chlorogenic acid content in our study. The MYB–bHLH–WD40 (MBW) complex is a well-known regulator of the phenylpropanoid pathway (Feller et al., 2011; Li, 2014). In tomato, *SlAN1* (WD40), for example, is an effective promoter of anthocyanin accumulation, directing pathway flux toward flavonol accumulation and acting in coordination with bHLH and MYB proteins (Gao et al., 2018). *bHLH153* was herein associated with chlorogenic acid content, pointing to a potential role in the phenylpropanoid pathway, particularly given its expression in leaves (Sun et al., 2015). Because a large proportion of plant dry matter is composed of polyphenol-derived lignin, MYB/SANT-like protein (*Solyc04g014855*) emerges as a promising candidate regulator of dry matter accumulation by promoting lignin deposition in cell walls (D’Esposito et al., 2019; Dong and Lin, 2021). Indeed, Yan et al. (2023) showed that it is expressed in leaves.

Genes encoding signaling proteins and growth regulators have also been identified as candidate genes associated with both dry matter and chlorogenic acid content in the ToMAGIC. Receptor-like protein kinases can sense external and internal signals and activate downstream signaling pathways, thereby mediating appropriate plant responses (Guo et al., 2009; Sakamoto et al., 2012; Wu et al., 2014). In Arabidopsis, *AtHERK1* and *AtTHESEUS1* are required for brassinosteroid-mediated cell elongation during vegetative growth (Guo et al., 2009). *SlHERK1* and *SlTHESEUS1* expression pattern in tomato tissues (Fei et al., 2011) may indicate a similar role. Moreover, brassinosteroids have been reported to crosstalk with the phenylpropanoid pathway, thereby promoting flavonoid accumulation under stress and contributing to ROS scavenging (Yaqoob et al., 2022). Likewise, *SlMAPKK1* (*Solyc12g009020*), associated with dry matter, has been linked to tomato development in response to hormonal, pathogenic, and environmental cues (Wu et al., 2014). Lastly, the auxin-responsive genes *GH3.8* (*Solyc07g053030*) and *GH3.15* (*Solyc12g005310*) genes have been implicated in reducing plant height, lateral root development and gravitropism in tomato (Sun et al., 2020; Ai et al., 2023). It is plausible that the auxin-responsive proteins identified in our study may affect the growth of ToMAGIC lines.

The low to moderate variance explained by GWAS in the traits we studied can be attributed to complex genetic architecture of these traits. Dry matter is highly polygenic and is influenced by numerous genes and regulatory factors that are regulated dynamically during plant development (de Oliveira Silva et al., 2018; D’Esposito et al., 2019). Previous studies have shown that leaf phenolic content is generally higher and phenolic profiles are more diverse than in fruits, stems, and roots (Minutolo et al., 2013; Larbat et al., 2014; Nunes-Nesi et al., 2019). At the same time, studies have shown that some leaf metabolites are more strongly influenced by genetic and environmental factors (Nunes-Nesi et al., 2019). This correlates well with the heritability results discussed earlier. Moreover, although higher values suggest more promising associations, low-effect variants may help identify minor-effect or epistatic alleles which are often missed because of limited mapping resolution or statistical power (Tibbs Cortes et al., 2021; Torgeman and Zamir, 2023).

Taken together, the genetic variation observed and the identification of favorable alleles provide promising opportunities for exploiting the ToMAGIC population in breeding programs aimed at improving tomato resilience. QTL pyramiding has shown to be an effective strategy for increasing phenolic content in tomato fruit (Sacco et al., 2013; Rigano et al., 2014). Since our candidate loci are distributed across several chromosomes, this may facilitate combining multiple favorable alleles within a single line (Rosa-Martínez et al., 2023). For instance, pyramiding of all six favorable alleles identified in this study could be achieved by crossing lines 763 (three favorable alleles) and 839 (four favorable alleles). Moreover, an examination of the top-ranked lines (**Supplementary Data Sheet 1**) for dry matter and metabolite content identified several additional ToMAGIC lines (e.g., 432, 496, 776, 796, and 832) carrying multiple favorable alleles, offering several crossing combinations to stack these alleles into a single genetic background within relatively few generations.

Moreover, the SPET marker panel described here is useful not only for diversity analysis and association studies, but also for applied breeding (Barchi et al., 2019). Because SNP positions and probe information are publicly available through NCBI BioProject PRJNA542237 (Barchi et al., 2019), informative loci can be converted into alternative genotyping formats better suited to routine breeding applications. For example, trait-associated SNPs identified with SPET could be converted into allele-specific assays such as KASP, PACE, or TaqMan to support marker-assisted selection. Furthermore, the availability of flanking sequence information facilitates the design of locus-specific primers and the selection of a smaller set of informative markers adapted to specific germplasm and breeding objectives. This enhances the practical utility of the dataset for breeding applications.

Some limitations of this study should be acknowledged. First, phenotyping was conducted in a single environment and growing season, which may limit the ability to fully capture genotype × environment interactions affecting metabolite accumulation. Second, the relatively small population size used for the metabolite GWAS (130 lines) may have reduced the power to detect loci with small effects. Additionally, the use of fixed genomic windows to define candidate regions may be less precise than linkage disequilibrium–based interval definition. Finally, further validation will be necessary to confirm the biological relevance of the identified loci, including expression analyses, functional validation through gene editing, and multi-environment evaluations to confirm the stability of the detected associations.

## 5 Conclusions

We quantified dry matter and polyphenols in the leaves of a large tomato MAGIC population. Our results revealed significant natural variation within the mapping population for dry matter and the contents of chlorogenic acid, rutin, quercetin, kaempferol, and total phenolics. We identified six genotype–phenotype associations that, after further analysis, enabled the prioritization of several candidate genes putatively involved in the control of these traits. Future transcriptomic studies and genome-editing approaches should shed light on the role of these candidate genes and provide insights into the complex nature of these traits. Another important outcome of this work is that the use of core subsets of this population proved useful for the genetic dissection of traits that require substantial investment for evaluation, without the need to analyze the entire population. In summary, this study provides a valuable resource for understanding dry matter and phenylpropanoid accumulation in tomato leaves, which may be of great interest for selecting stress-tolerant tomato varieties and for the development of climate-smart cultivars requiring fewer inputs for more sustainable tomato production.

## Data Availability

Raw data supporting the conclusions of this article will be made available by the authors upon request.
