# Supplementary material for: A tomato MAGIC population reveals candidate genes for leaf dry matter and phenolics, two key traits for stress resilience and climate-smart breeding
Source: Front Plant Sci. 2026 May 5;17:1765593. doi: 10.3389/fpls.2026.1765593 (PMC13183629; doi:10.3389/fpls.2026.1765593)
Supplement: Supplementary file 1 [file DataSheet1.pdf]

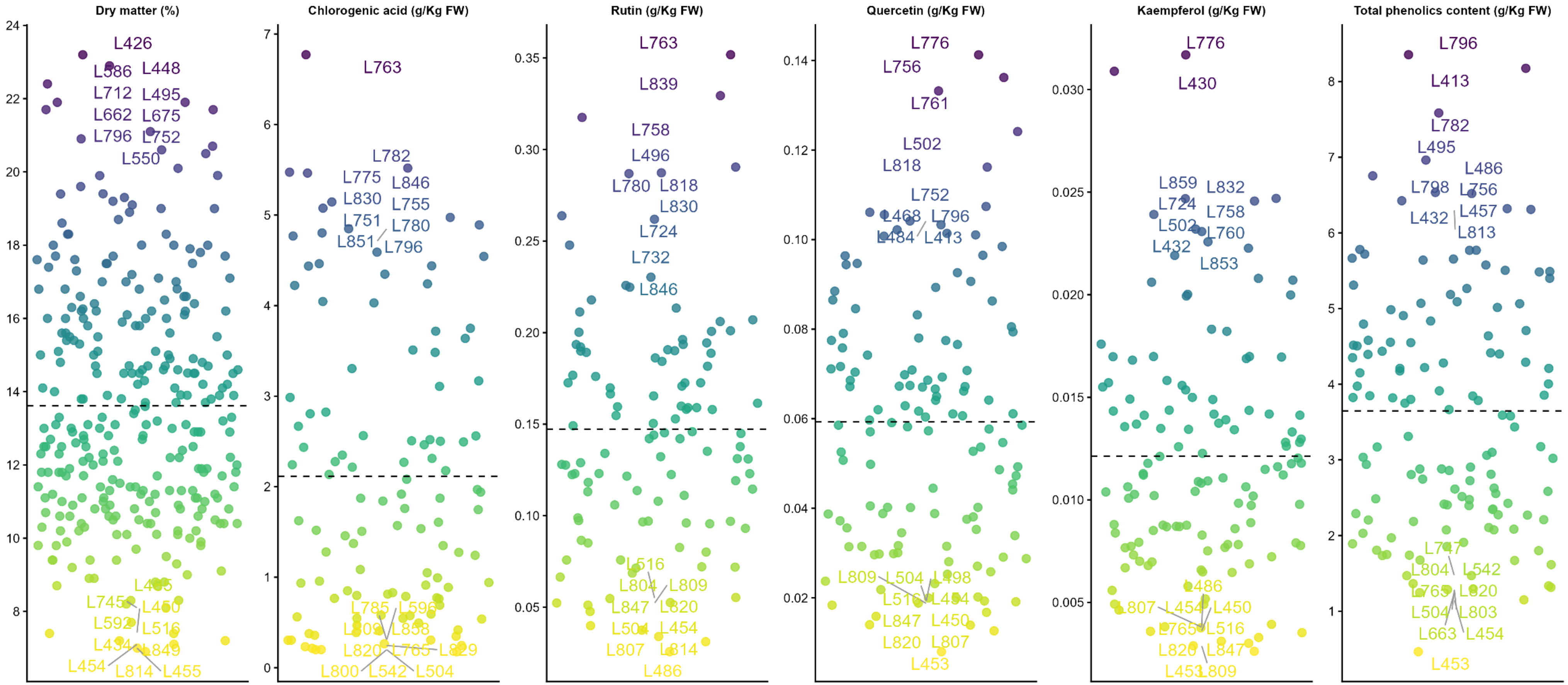

Supplementary data 1 - Variation across ToMAGIC lines for dry matter (293 lines) and phenolic compounds (130 lines) in young leaves. Each panel represents one trait: Dry matter (%), Chlorogenic acid (g kg^{-1} FW), Rutin (g kg^{-1} FW), Quercetin (g kg^{-1} FW), Kaempferol (g kg^{-1} FW), and Total phenolics content (g kg^{-1} FW). Each dot corresponds to the mean of a ToMAGIC line, colored according to the magnitude of the trait using the viridis color scale (Yellow = low values, Purple = high values). The dashed horizontal line indicates the overall mean for the corresponding trait. The ten lines with the highest and lowest values are labeled to highlight genotypes with extreme phenolic or dry matter accumulation profiles.
