# Supplementary material for: A tomato MAGIC population reveals candidate genes for leaf dry matter and phenolics, two key traits for stress resilience and climate-smart breeding
Source: Front Plant Sci. 2026 May 5;17:1765593. doi: 10.3389/fpls.2026.1765593 (PMC13183629; doi:10.3389/fpls.2026.1765593)
Supplement: Supplementary file 2 [file DataSheet2.pdf]

Supplementary data 2 – Quantile–quantile (QQ) plots showing model fit for different GWAS approaches applied to each one of the evaluated traits in the ToMAGIC population. Each panel represents the distribution of observed versus expected $–\log_{10}(p)$ values for the traits Dry matter (%; dw), Chlorogenic acid (g kg^{-1} FW; cg), Rutin (g kg^{-1} FW; rut), Quercetin (g kg^{-1} FW; quer), Kaempferol (g kg^{-1} FW; k), and Total phenolics content (g kg^{-1} FW; tpc) in function of the distinct GWAS models used, namely GLM, MLM, CMLM, MLMM, and BLINK. The diagonal line represents the null hypothesis of no association. Deviations from the line at higher $–\log_{10}(p)$ values indicate potential true marker–trait associations, while proximity to the line reflects adequate control of false positives and model fit.

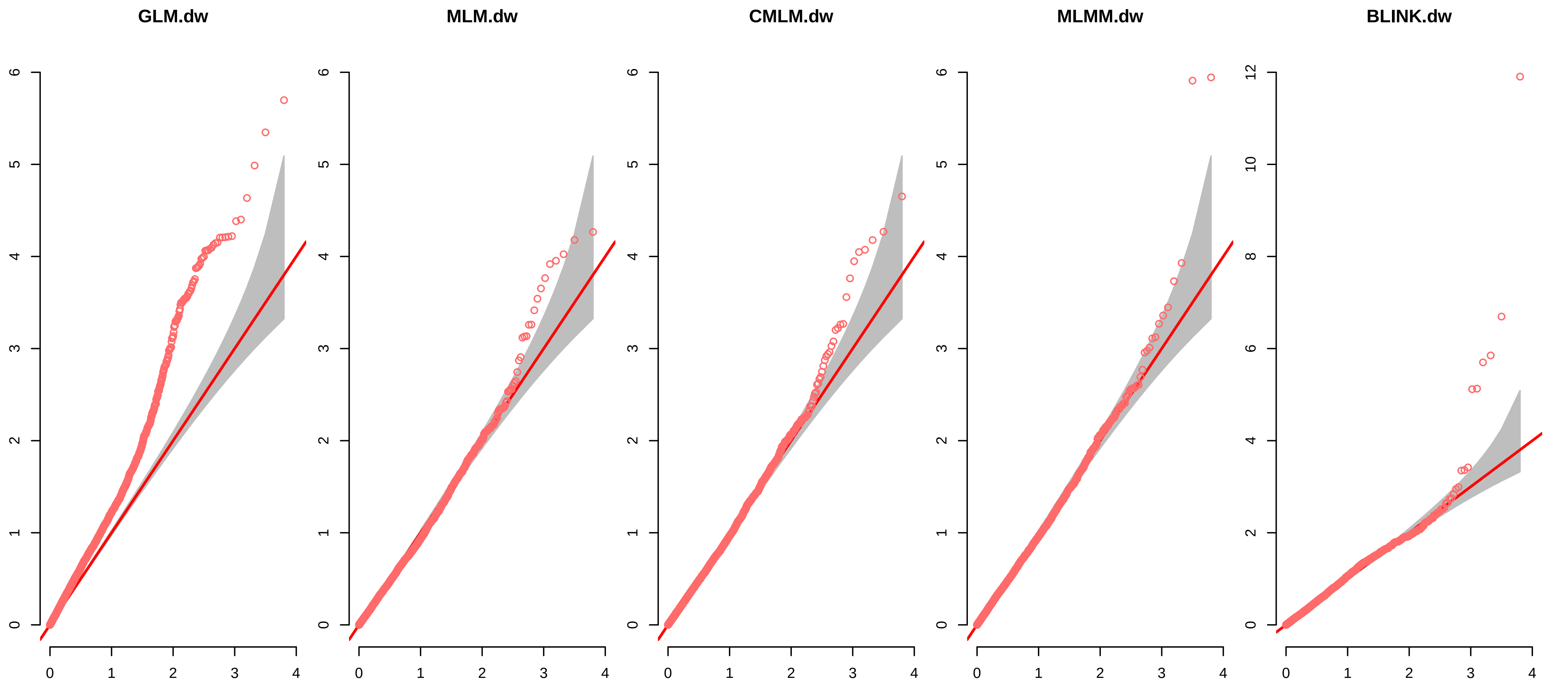
GLM.dw
MLM.dw
CMLM.dw
MLMM.dw
BLINK.dw
0
1
2
3
4
5
6
8
10
12

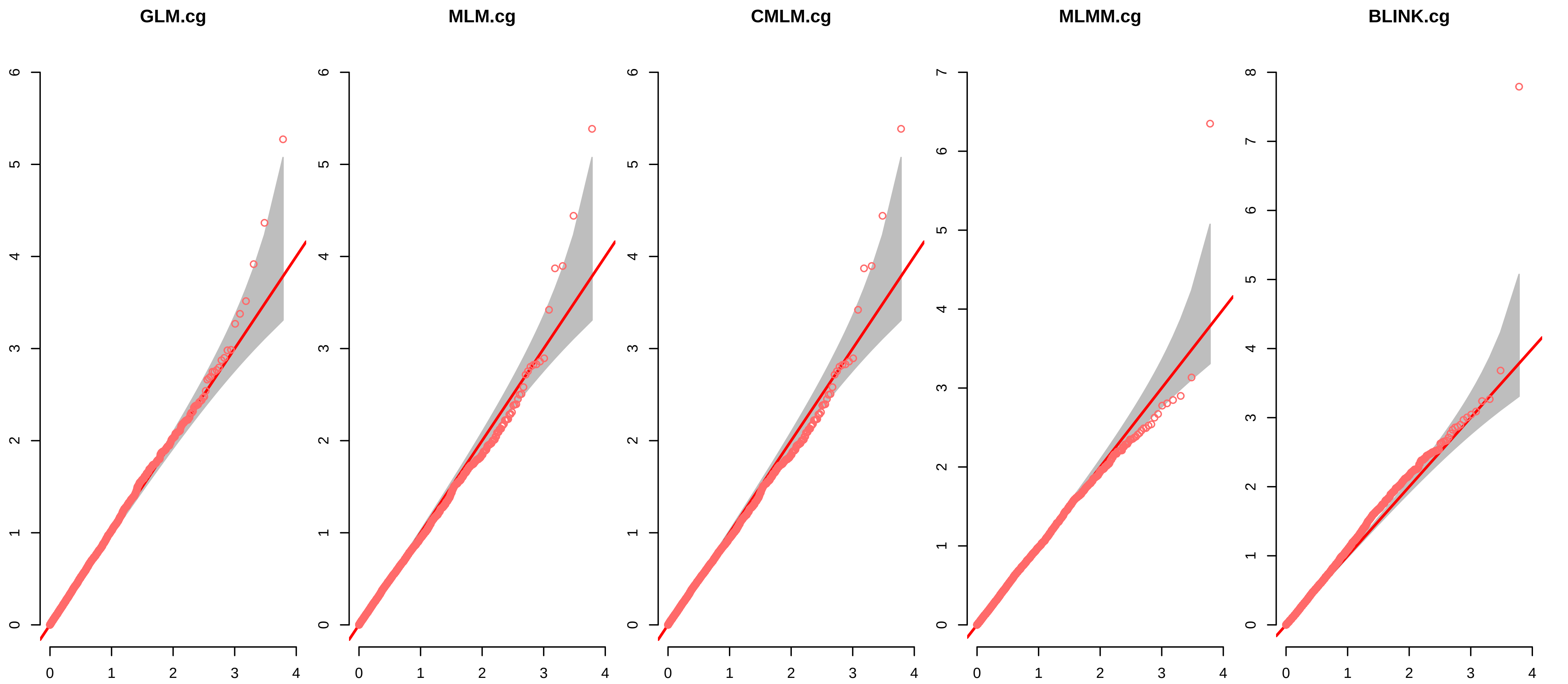
GLM.cg
MLM.cg
CMLM.cg
MLMM.cg
BLINK.cg
0
1
2
3
4
5
6
7
8

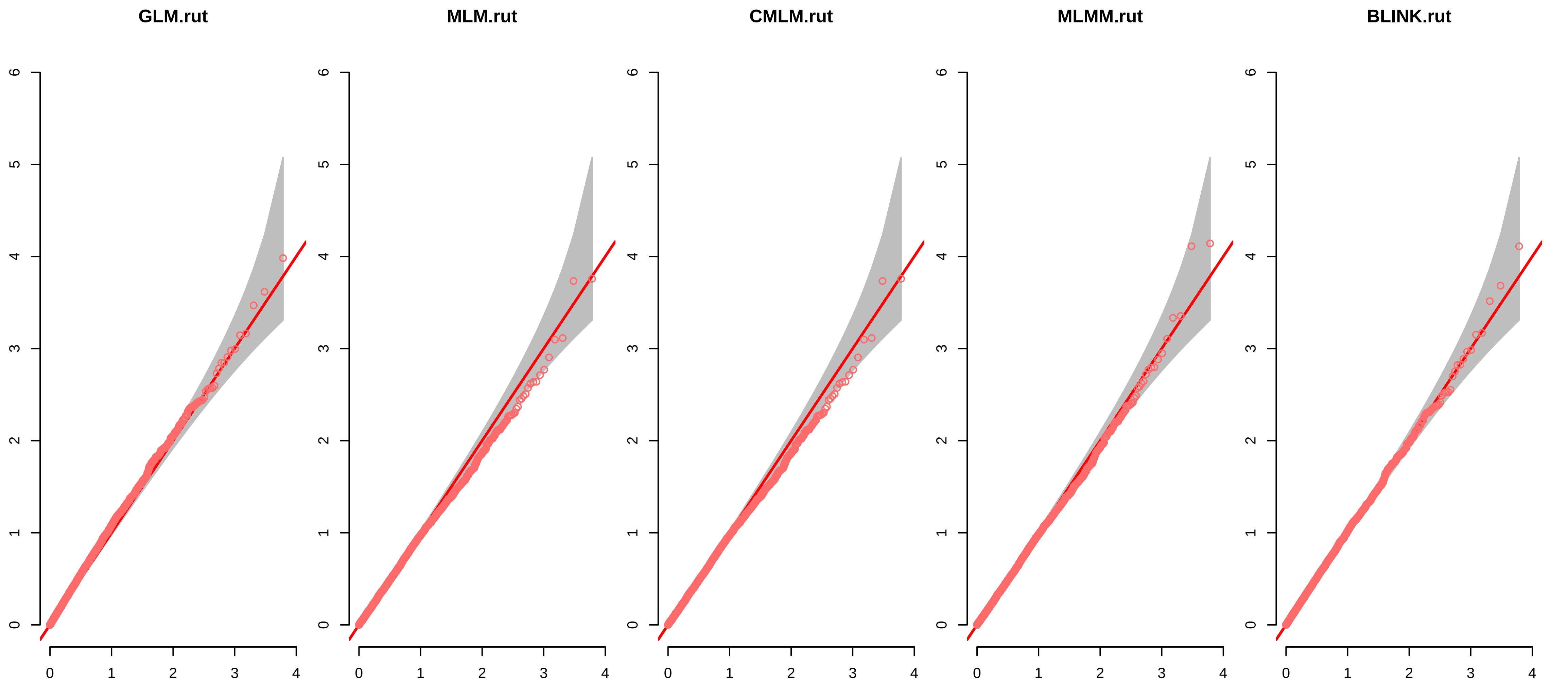
GLM.rut
MLM.rut
CMLM.rut
MLMM.rut
BLINK.rut
0
1
2
3
4
5
6
0
1
2
3
4

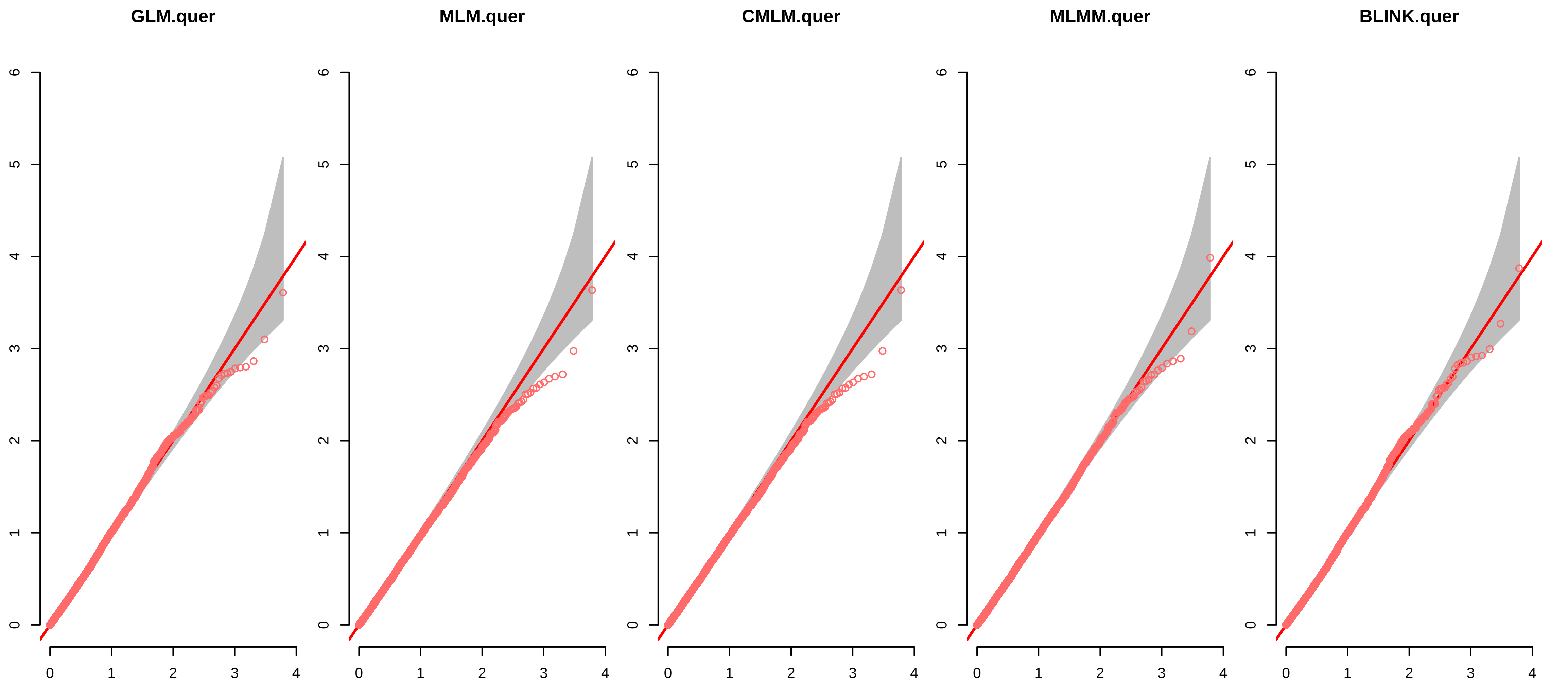
GLM.quer
MLM.quer
CMLM.quer
MLMM.quer
BLINK.quer
0
1
2
3
4
5
6
0
1
2
3
4

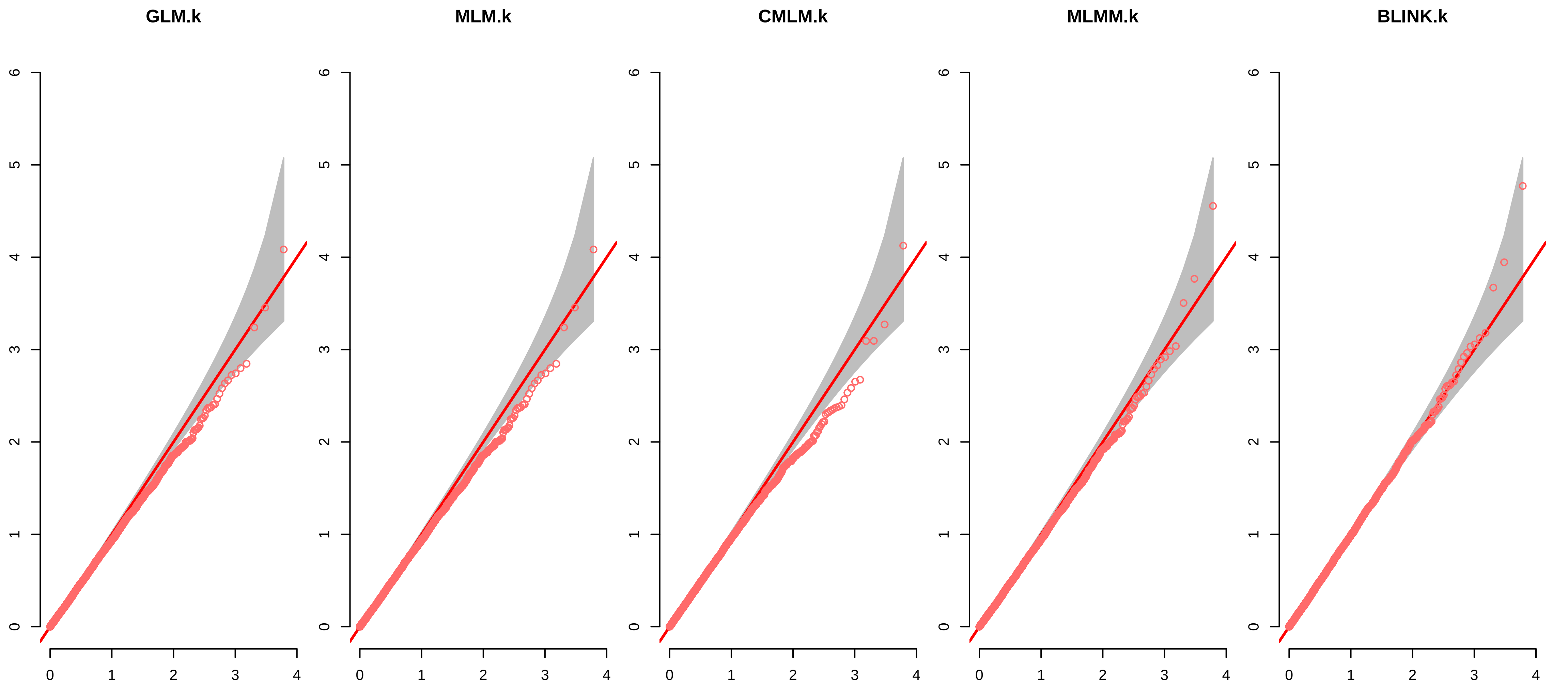
GLM.k
MLM.k
CMLM.k
MLMM.k
BLINK.k
0
1
2
3
4
5
6
0
1
2
3
4

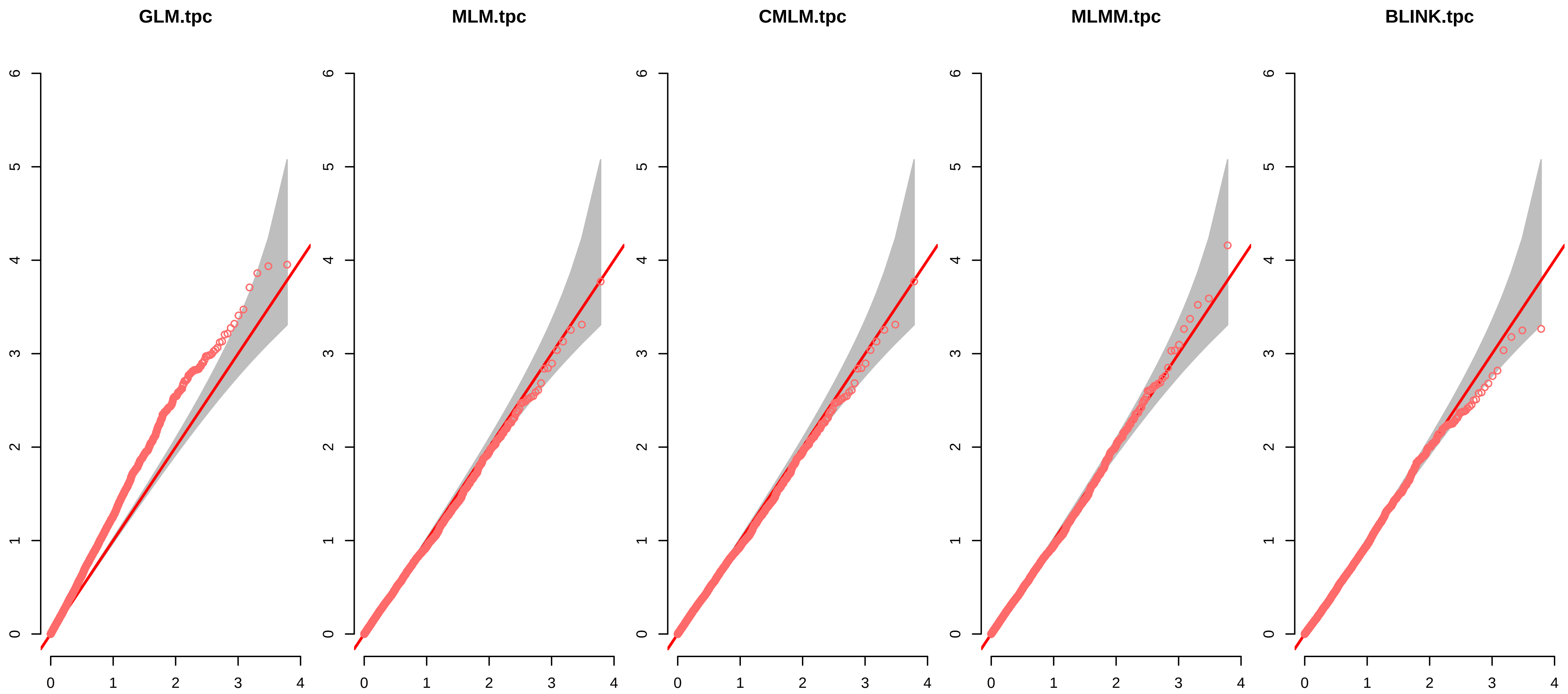
GLM.tpc
MLM.tpc
CMLM.tpc
MLMM.tpc
BLINK.tpc
0
1
2
3
4
5
6
0
1
2
3
4
