# Supplementary material for: A tomato MAGIC population reveals candidate genes for leaf dry matter and phenolics, two key traits for stress resilience and climate-smart breeding
Source: Front Plant Sci. 2026 May 5;17:1765593. doi: 10.3389/fpls.2026.1765593 (PMC13183629; doi:10.3389/fpls.2026.1765593)

**Supplementary Data 3 –** Manhattan plots of GWAS results for rutin (g $kg^{-1}$ FW; rut), quercetin (g $kg^{-1}$ FW; quer), kaempferol (g $kg^{-1}$ FW; k), and total phenolic content (g $kg^{-1}$ FW; tpc) based on BLINK model results. Each panel displays the distribution of observed $-\log_{10}(p)$ values for SNPs tested across the 12 tomato chromosomes. These traits were not included in the main manuscript due to the absence of significant associations. SNPs closest to the significance threshold are highlighted in green. ANOVA indicated a significant effect of genotype for all traits except rutin and kaempferol.

# Manhattan plot of GWAS results for rutin (rut) content (g kg$^{-1}$ FW)

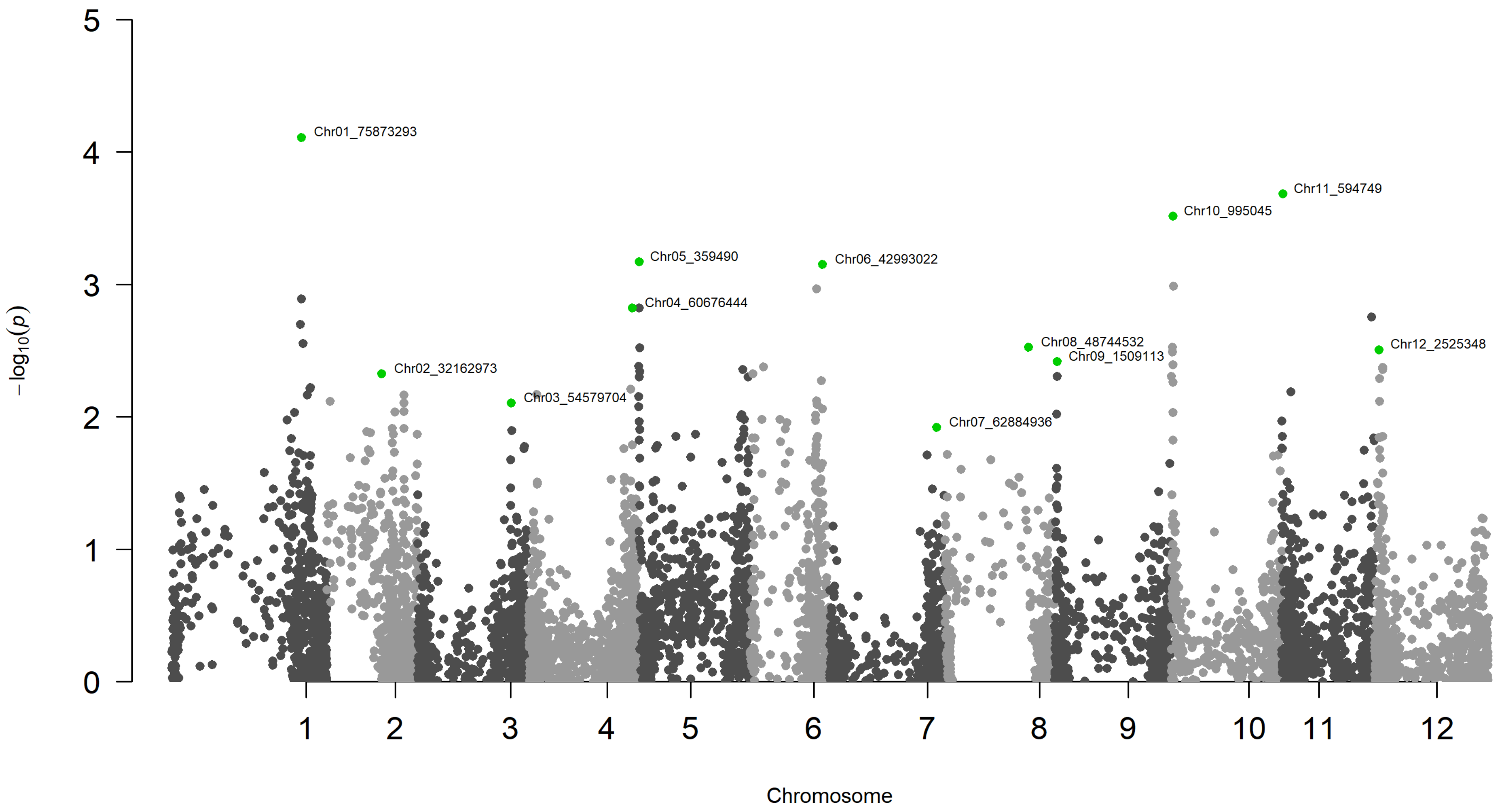

Manhattan plot of GWAS results for quercetin content (g kg$^{-1}$ FW)

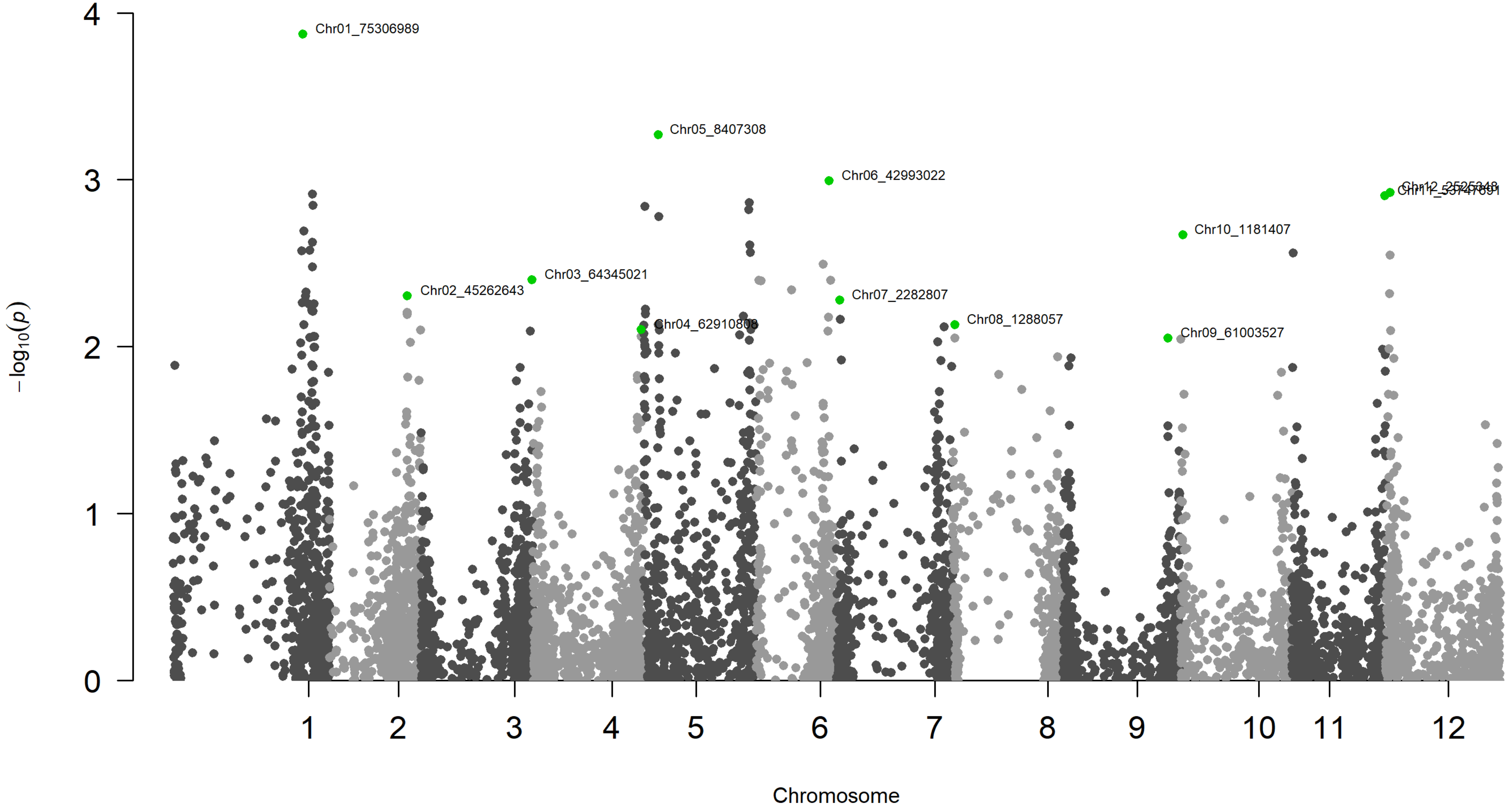

Manhattan plot of GWAS results for kaempferol (k) content (g kg$^{-1}$ FW)

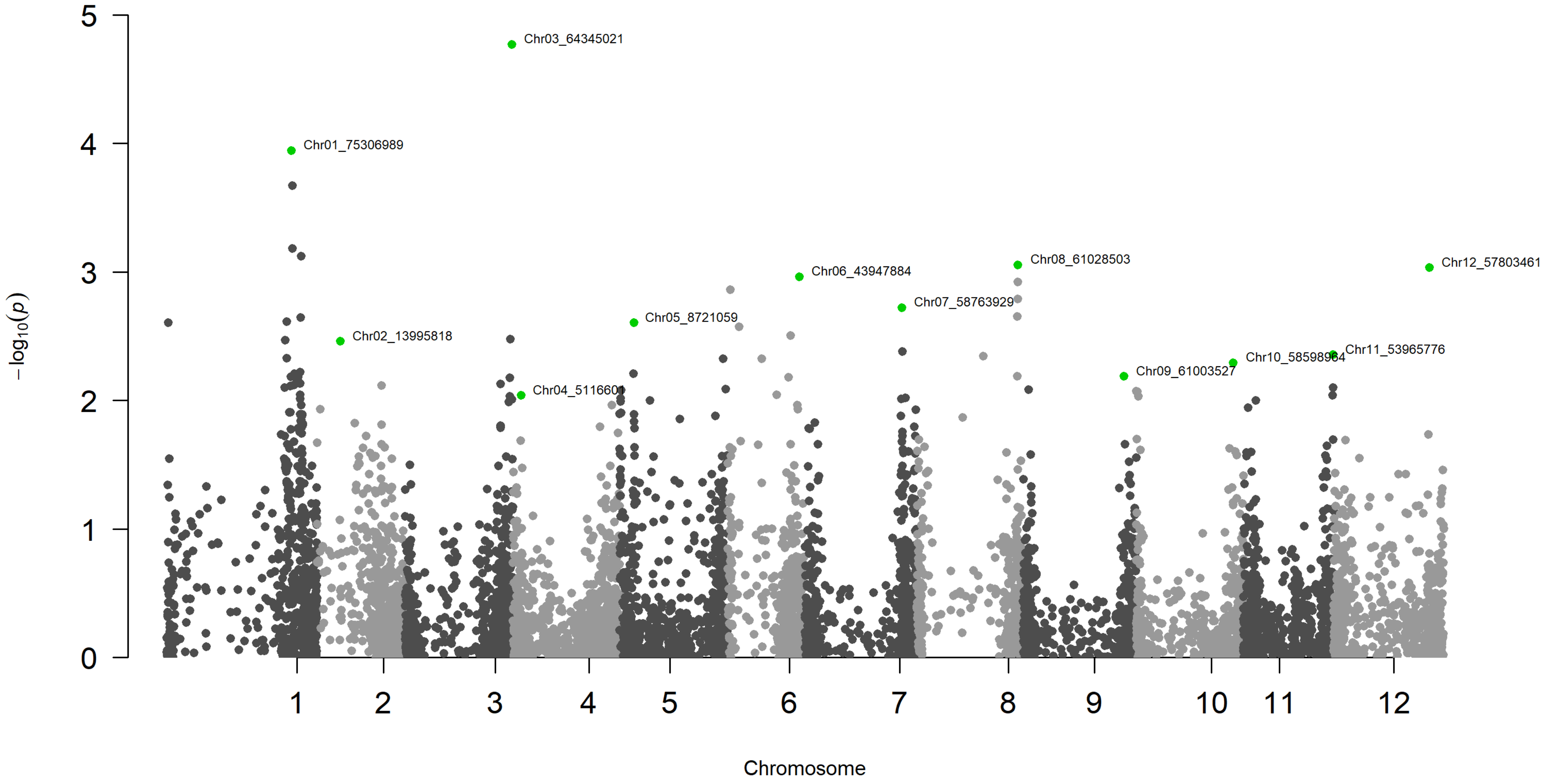

Manhattan plot of GWAS results for total phenolic content (tpc) content (g kg$^{-1}$ FW)

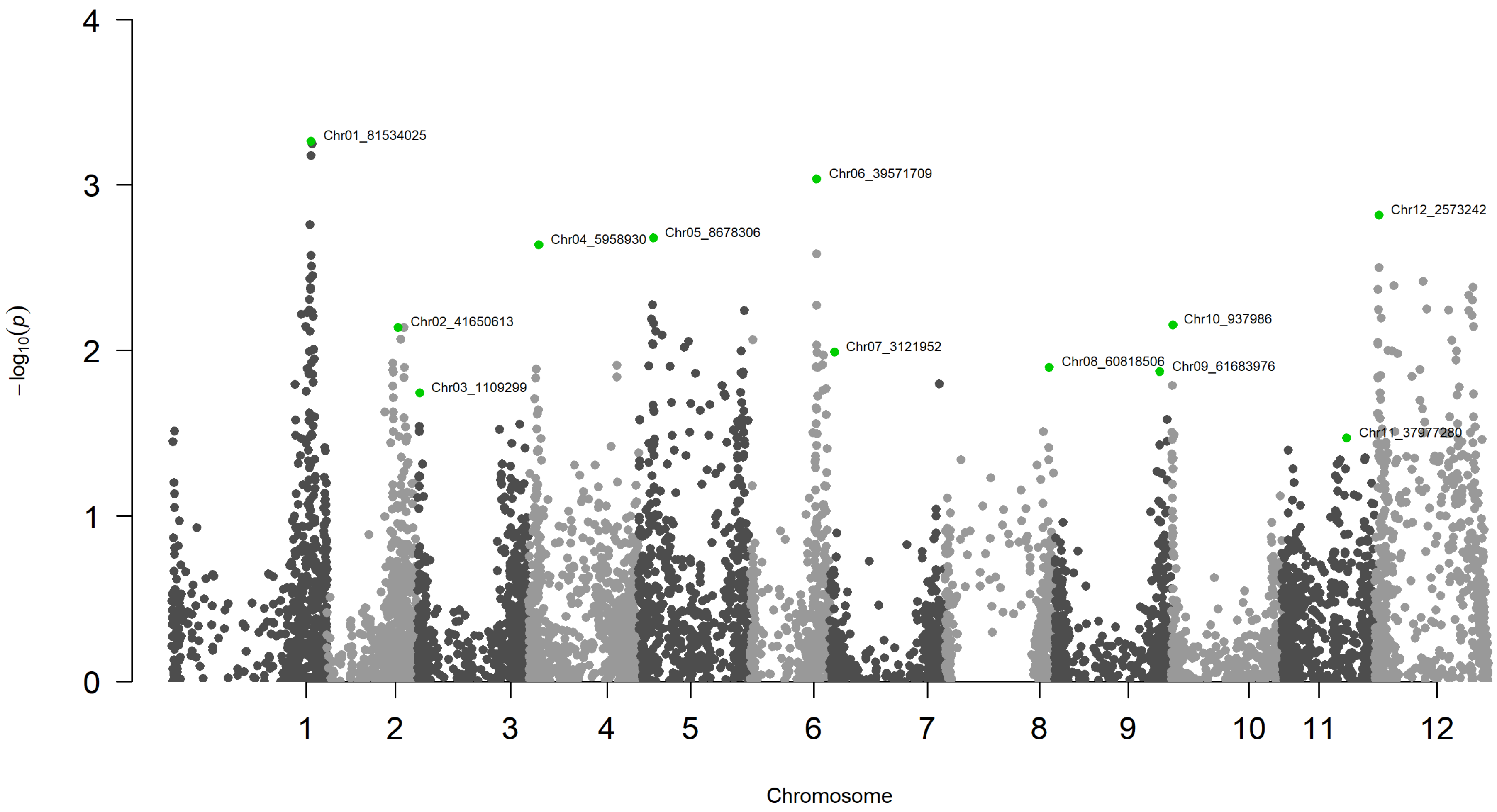

Supplement: Supplementary file 3 [file DataSheet3.pdf]
