# Supplementary material for: A tomato MAGIC population reveals candidate genes for leaf dry matter and phenolics, two key traits for stress resilience and climate-smart breeding
Source: Front Plant Sci. 2026 May 5;17:1765593. doi: 10.3389/fpls.2026.1765593 (PMC13183629; doi:10.3389/fpls.2026.1765593)
Supplement: Supplementary file 4 [file DataSheet4.pdf]

Supplementary data 4 - Presence of SnpEff-predicted high-impact variants in candidate gene sequences across ToMAGIC parental lines

This information serves as a complement to the results presented in Section 3.3, "Genome-Wide Association Study (GWAS)," by adding parent-specific variant details and clarifying the criteria used to prioritize candidate genes.

Candidate genes were prioritized based on their physical proximity to significant SNPs within a ±100 kb physical window. Gene prioritization was based on the presence of SnpEff-predicted high-impact variants, including frameshift mutations, stop-gained variants, splice-site alterations, and premature start codon gains segregating among the ToMAGIC parental lines. Functional annotation related to plant development, transcriptional regulation, metabolite transport, and hormone signaling were also considered to support their potential biological relevance.

Chromosome 1 locus closest candidate gene corresponded to a *Gibberellin receptor* (*Solyc01g098390*). Additional candidates included *LOB domain-containing protein 20* (*Solyc01g098220*), a *FRIGIDA-like protein* (*Solyc01g098240*), and two *Sugar Facilitator Proteins* (*Solyc01g098490* and *Solyc01g098500*). These genes harbor high-impact mutations, including stop-gained, premature start codon gain, and frameshift variants. Notably, parent LA2251 (SLC2) carried high-impact variants affecting the *LOB domain-containing protein 20* and *Sugar Facilitator 5*, while the four *S. pimpinellifolium* parents (SP1 to SP4) shared a variant in *FRIGIDA-like*. Additionally, BGV006454 (SP2) and BGV007145 (SP1) carried disruptive variants in both Sugar Facilitator genes.

The association found in chromosome 4 was located within *GRAS transcription factor* (*Solyc04g014830*) exonic sequence. The missense variant may have a non-disruptive but potentially functional effect on the coded protein. Moreover, this candidate harbored two other high-impact mutations (frameshift variants) in its coding sequence in *S. lycopersicum* var. *cerasiforme* parental lines BGV007931 (SLC1) and LA2251 (SLC2), which could explain the trait variation among lines. Notwithstanding, we found other plausible candidates in this physical window, namely a *MYB/SANT-like Protein* (*Solyc04g014855*). It showed a frameshift mutation in parent BGV007145 (SP1).

In chromosome 5, alternative candidate gene to SNP 61,754,534 *Major Facilitator Superfamily protein* (*Solyc05g051920*) carried two frameshift mutations in parents BGV007145 (SP1) and BGV013720 (SP4). Contrastingly, all four *S. pimpinellifolium* parents carried an allele found in the intronic region of *Sulfate Transporter* (*Solyc05g054740*) with no clear impact on protein function. However, ToMAGIC *S. pimpinellifolium* parents BGV006454 (SP2) and BGV013720 (SP4) presented a splice acceptor variant, potentially leading to loss of function or aberrant proteins, in *Solyc05g054740 sequence*. Near to this position, *Zinc Finger Protein* (*Solyc05g054650*) of parent LA2251 (SLC2), *β-hexosaminidase* (*Solyc05g054710*) of parents BGV006454 (SP2) and BGV015382 (SP3), and *HERK 1 Protein Kinase* (*Solyc05g054860*) of BGV006454 (SP2) showed at least one variant that typically causes severe disruptions to gene function that could be segregating among the population and causing major differences in the trait.

Chromosome 12 SNP located within the coding sequence of *WD40 Transcription Factor* (*Solyc12g009030*) represented a missense variant in parent BGV007145 (SP1). Upstream-located *Cytokinin dehydrogenase 5* (*Solyc12g008920*) was also considered a strong candidate due to the presence of splice-site variants in parents BGV007145 (SP1), BGV013720 (SP4), and LA2251 (SLC2).

Finally, chromosome 10 locus was associated with chlorogenic acid content. *Auxin-responsive GH3 Family Proteins* (*Solyc10g006610*, *Solyc10g006615*, and *Solyc10g006630*), *bHLH Transcription Factor 153* (*Solyc10g006640*), *Quinone reductase* (*Solyc10g006650*), and *THESEUS 1 Protein Kinase* (*Solyc10g006870*) were identified as additional candidates. These encompass at least one high-impact mutation among the ToMAGIC parents putatively affecting accumulation of this phenolic in the tomato leaves. For instance, parent BGV006454 (SP2) had frameshift mutations in *Solyc10g006610*, *Solyc10g006615*, and *Solyc10g006640* sequences. Similarly, BGV007145 (SP1), BGV013720 (SP4), and LA2251 (SLC2) presented stop gained variants in *Solyc10g006630*, *Solyc10g006650*, and *Solyc10g006870*, respectively, potentially affecting chlorogenic acid accumulation.
